# Optimization for maximum specific energy density of a lithium-ion battery using progressive quadratic response surface method and design of experiments

**DOI:** 10.1038/s41598-020-72442-4

**Published:** 2020-09-24

**Authors:** Ji-San Kim, Dong-Chan Lee, Jeong-Joo Lee, Chang-Wan Kim

**Affiliations:** 1 Graduate School of Mechanical Design and Production Engineering, Konkuk University, 120, Neung dong-ro, Gwangjin-gu, Seoul, 05029 Republic of Korea; 2 School of Mechanical Engineering, Konkuk University, 120, Neung dong-ro, Gwangjin-gu, Seoul, 05029 Republic of Korea

**Keywords:** Batteries, Mechanical engineering, Batteries

## Abstract

The demand for high-capacity lithium-ion batteries (LIB) in electric vehicles has increased. In this study, optimization to maximize the specific energy density of a cell is conducted using the LIB electrochemical model and sequential approximate optimization (SAO). First, the design of experiments is performed to analyze the sensitivity of design factors important to the specific energy density, such as electrode and separator thicknesses, porosity, and particle size. Then, the design variables of the cell are optimized for maximum specific energy density using the progressive quadratic response surface method (PQRSM), which is one of the SAO techniques. As a result of optimization, the thickness ratio of the electrode was optimized and the porosity was reduced to keep the specific energy density high, while still maintaining the specific power density performance. This led to an increase in the specific energy density of 56.8% and a reduction in the polarization phenomenon of 11.5%. The specific energy density effectively improved through minimum computation despite the nonlinearity of the electrochemical model in PQRSM optimization.

## Introduction

Due to their high theoretical energy density and long life, lithium-ion batteries (LIB) are widely used as rechargeable batteries. The demand for high-power, high-capacity LIB has witnessed a surge due to the increasing demand for electric vehicles and energy storage devices^1–3^. To cater to this trend, the energy density of LIB must be improved. For this, new electrode materials are being researched and developed. However, the development of new electrode materials requires significant time and effort; as such, many researchers are currently conducting studies on the same.

Therefore, one way to reduce the cost of research and development is to optimize the design variables of existing electrode materials, such as porosity and thickness, for enhanced power and capacity of LIB^4–15^. It is crucial to optimize the design variables to reach the target performance because power and capacity share a tradeoff relationship. However, the relationship between design variables and the performance of lithium-ion batteries is highly nonlinear; therefore, it is difficult to design them through experiments. To overcome these difficulties, optimization using numerical models that consider electrochemical reactions is employed, which is an effective method. Recent studies have been conducted to optimize cell design variables using numerical models for the design of high-power/high-capacity batteries^4^.

Previously, Newman conducted a parametric study using a Ragone plot to maximize the specific energy density of the battery^5–11^. A Ragone plot is a simple graph that shows the relationship between the specific energy and the specific power of a cell. Doyle et al. developed an electrochemical model for predicting the charge and discharge performance of a battery using the porous electrode theory and the concentrated solution theory. This formed the basis for later research on LIB optimization^5^. Through a parametric study, Doyle and Newman compared the specific energy density of cells consisting of various electrode thicknesses, porosities, and electrolytes, and proposed an optimized cell using a Ragone plot^6–8^. Srinivasan and Newman optimized the porosity and thickness of a positive electrode for various C-rates while maintaining the capacity ratio of the two electrodes, the thickness and porosity of the separator, and the porosity of the negative electrode^9^. Christensen et al. optimized the thickness and porosity of lithium titanate (LTO) negative electrodes for electric vehicles and used a Ragone plot to predict the power performance^10^. Stewart et al. improved a Ragone plot considering the pulse performance of a hybrid electric vehicle (HEV) and optimized the specific power-to-energy ratio of the HEV’s battery cell^11^. Appiah et al. optimized the thickness and porositiy of LiNi_0.6_Co_0.2_Mn_0.2_O_2_ cathode through a parametric study using Ragone plot^12^. However, derivation of the optimal variables using a Ragone plot and parametric study can be computationally expensive; as such, research using numerical optimization techniques is needed.

For example, Xue et al. selected 12 design variables, including electrode porosity, diffusion coefficient, and various C-rates and calculated the gradient through the complex-step approximation method. They then optimized the specific energy density using sequential quadratic programming methods^13^. Golmon et al. developed a multiscale battery model that additionally considered the microscale, used an adjoint sensitivity analysis to calculate the gradient, and optimized capacity of the battery^14^. Changhong Liu and Lin Liu optimized the capacity loss of the battery using a gradient-based algorithm called multiple starting point search and improved the capacity loss of the cell by 22%^15^. However, gradient-based optimization is a complex process that requires various steps of computation and time. Moreover, it is sensitive to numerical noise, and the optimization results converge on a local optimum^16^.

To avoid the disadvantages of gradient-based optimization, researchers have studied many algorithms that do not require gradient calculation^17–19^. Among them, the progressive quadratic response surface method (PQRSM) is one of the sequential approximate optimization (SAO) techniques that can be effectively applied to nonlinear problems without gradient calculations^20^. Furthermore, PQRSM applies a trust region algorithm that guarantees weak global convergence and has a low probability of convergence on a local optimum^21–23^. In addition, unlike the parametric study using a Ragone plot, which requires hundreds of simulations to analyze a single cell, the PQRSM requires fewer calculations for optimal results. For these advantages, PQRSM has used in various engineering fields; however, it has never been applied to the optimization of a LIB^24,25^.

In this study, optimization for the maximum specific energy density of a LIB cell is performed using design of experiments, the PQRSM, and an electrochemical model of the LIB that is used to calculate the specific energy density and the specific power density. First, design of experiments (DOE) was conducted to analyze the sensitivity of eight cell design factors, including anode thickness, cathode thickness, separator thickness, anode porosity, cathode porosity, separator porosity, anode particle size, and cathode particle size. Design factors sensitive to specific energy density and specific power density were selected as design variables through a sensitivity analysis of DOE. The PQRSM, which guarantees the weak global convergence and does not require gradient calculation, was used as the optimization algorithm to maximize the specific energy density of LIB. After optimization, the differences in the specific energy density and specific power density of the initial and optimized cell were compared through constant current discharge. It verified the superiority of the optimized design result.

## Electrochemical lithium-ion battery model

**Figure 1:**
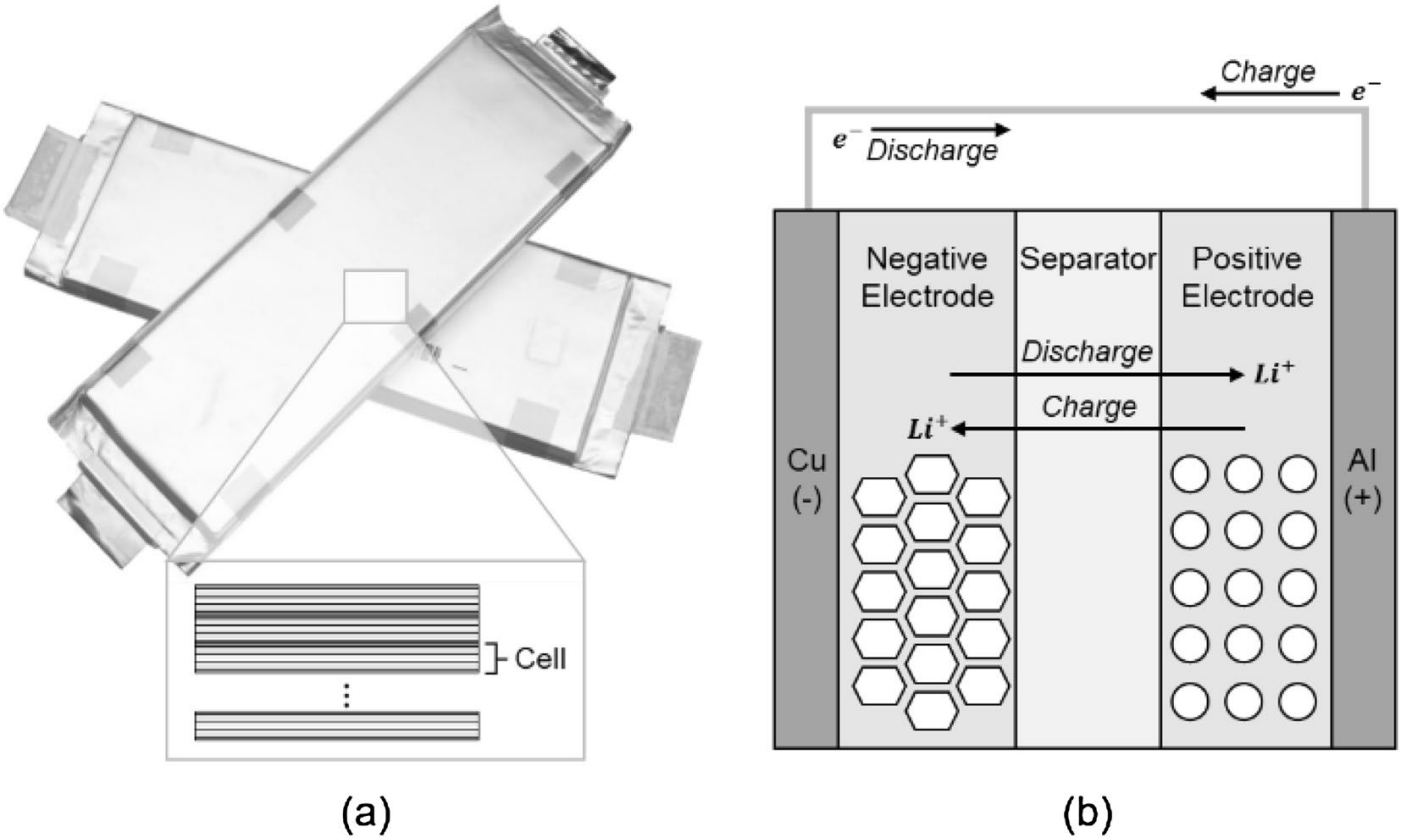
Schematic of (**a**) LIB pouch cell and (**b**) LIB electrochemical model.

The LIB model uses the pseudo-two-dimensional (P2D) model developed by Doyle et al., which applies the porous electrode theory and the concentrated solution theory^5,6^. The LIB cell consisted of 5 layers, including collector, electrode, and separator, and used graphite/LMO-type cells. Figure 1 shows the structure of the LIB model and a schematic of the charge and discharge cycle.

Equations (1)–(5) are the governing equations of the Li-ion cell electrochemical reaction. Equation (1) is the electronic charge balance equation and Eq. (2) is the ionic charge balance in the electrolyte. Equation (3) is a form of Fick’s second law in spherical coordinates; it shows the diffusion of Li in the active particles. Equation (4) is the Li-ion ionic charge transport in the electrolyte. Equation (5) is the Li^+^ flux and Butler–Volmer equation on the active particle surface; it shows the defined reaction kinetics.

1 $$\nabla \cdot \left( {k_{1} \nabla \phi_{1} } \right) = - a \cdot j_{n}$$

2 $$\nabla \cdot \left( { - k_{2}^{eff} \nabla \phi_{2} + \frac{{2R_{c} Tk_{2}^{eff} }}{F}\left( {1 + \frac{{\partial \ln f_{ \pm } }}{{\partial \ln c_{2} }}} \right)\left( {1 - t_{ + } } \right)\nabla \left( {\ln c_{2} } \right)} \right) = a \cdot j_{n}$$

3 $$\frac{{dc_{1} }}{dt} + \frac{1}{{r^{2} }}\frac{\partial }{\partial r}\left( { - r^{2} D_{1} \frac{{\partial c_{1} }}{\partial r}} \right) = 0$$

4 $$\varepsilon \frac{{dc_{2} }}{dt} = \nabla \cdot \left( {D_{2}^{eff} \nabla c_{2} } \right) - \frac{1}{F}i_{2} \cdot \nabla t_{ + } + a \cdot j_{n} \left( {1 - t_{ + } } \right)$$

5 $$N_{0} = \frac{{ - j_{n} }}{F},\quad j_{n} = i_{0} \left\{ {\exp \left( {\frac{\eta F}{{R_{c} T}}} \right) - \exp \left( {\frac{{\left( { - \eta } \right)F}}{{R_{c} T}}} \right)} \right\}$$

**Figure 2:**
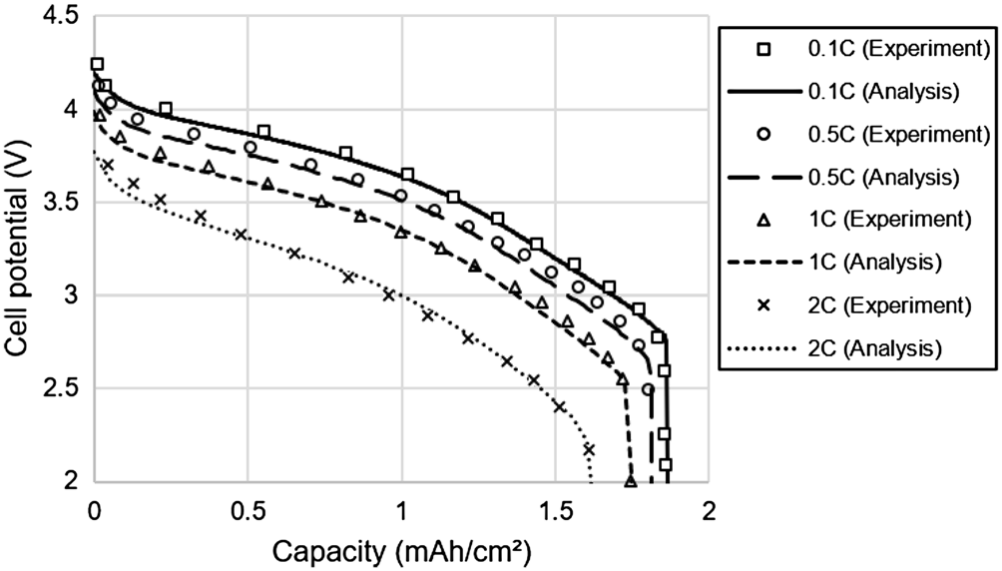
Comparison of discharge curves of the LIB cell between experiment^8^ and analysis.

The LIB electrochemical model was validated through comparisons with the discharge curves of Newman’s experiment. Figure 2 shows the experiment with 0.1C–2C discharge of the LIB cell and the discharge curves from the numerical model analysis. For the cell parameters, we referred to the LIB model by Newman and Doyle. Table 1 shows the cell parameters^6,8^. Collector parameters were referenced from a published paper^26^.

**Table 1:** Material properties and dimensions of the LIB cell^8,26^.

Parameter	Li_x_C_6_	LiMn_2_O_4_	Separator	Electrolyte	Al foil	Cu foil
Thickness (μm)	100	174	52	–	15	10
Porosity	0.357	0.444	0.46	–	–	–
Particle size (μm)	12.5	8.5	–	–	–	–
Density (kg/m^3^)	2,270	4,140	900	1,210	2,700	8,700
Diffusivity (m^2^/s)	3.9 × 10^−14^	1.0 × 10^−13^	–	7.5 × 10^−11^	–	–
Reaction rate constant	2.0 × 10^−11^	2.0 × 10^−11^	–	–		–
Electrical conductivity (S/m)	100	3.8	–	–	–	
Max. concentration (mol/m^3^)	26,390	22,860	–	–	–	–
Initial concentration (mol/m^3^)	22,055	4,000	–	1,000	–	–
Open circuit potential (V)	$$\begin{aligned} U_{n} & = 0.6379 + 0.5416\exp \left( { - 305.5309\theta_{n} } \right) + 0.044\tanh \left[ { - \frac{{\theta_{n} - 0.1958}}{0.1088}} \right] \\ & \quad - 0.1978\tanh \left[ {\frac{{\theta_{n} - 1.0571}}{0.0854}} \right] - 0.6875\tanh \left[ {\frac{{\theta_{n} + 0.0117}}{0.0529}} \right] - 0.0175\tanh \left[ {\frac{{\theta_{n} - 0.5692}}{0.0875}} \right] \\ U_{{\text{p}}} & = 4.1983 + 0.05656\tanh \left[ { - 14.5546\theta_{p} + 8.6094} \right] - 0.0275\left[ {\frac{1}{{\left( {0.9984 - \theta_{p} } \right)^{0.4925} }} - 1.9011} \right] \\ & \quad - 0.1571\exp \left( { - 0.0473\theta_{p}^{8} } \right) + 0.8102\exp \left[ { - 40\left( {\theta_{p} - 0.1339} \right)} \right] \\ \theta_{k} & = \frac{{c_{2,k} }}{{c_{2,k,max} }};\quad \left( {k = p,n} \right) \,state\,of\,charge\, \left( {SOC} \right) \\ \end{aligned}$$					

To confirm the initial specific energy density and specific energy density of the cell, constant current discharge was performed from 1 to 10C. The cell was discharged from the initial voltage of 4.2 V to the cut off voltage of 3 V. The 1C-rate current density was 25 A/m^2^ and the cell temperature is 298 K. The initial Li-ion concentration of the negative electrode was 22,055 mol/m^3^, and that of the positive electrode was 4,000 mol/m^3^. The initial concentration of the electrolyte was 1,000 mol/m^3^, and the rapid reduction in Li-ion concentration of the electrolyte could be prevented even at high current densities. In Eq. (6), the specific energy density was calculated by dividing the energy discharged up to the cut-off voltage by the cell mass. The specific power density was calculated through Eq. (7) by dividing the average power up to the cut-off voltage by cell mass. The mass of the cell was calculated by the sum of the weights of the anode, cathode, separator, electrolyte, and collector through Eq. (8).

6 $$E_{cell} = \frac{1}{{M_{cell} }}\mathop \smallint \limits_{0}^{{t_{end} }} V_{cell} \cdot i_{app} dt$$

7 $$P_{cell} = \frac{1}{{M_{cell} }}\mathop \smallint \limits_{0}^{{t_{end} }} V_{cell} \cdot i_{app} dt \cdot \frac{1}{{t_{end} }}$$

8 $$M_{cell} = M_{pos} + M_{neg} + M_{sep} + M_{electrolyte} + M_{collector}$$

**Figure 3:**
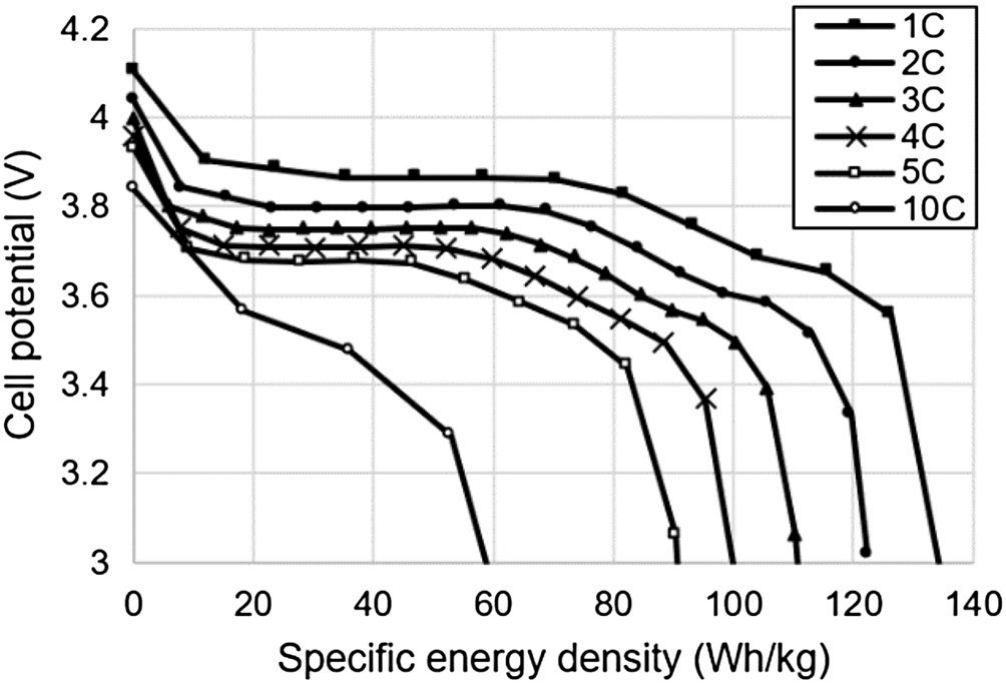
Discharge curves under various C rates for initial cell.

Figure 3 shows the results of constant current discharge from 1 to 10C. The cell exhibited a specific energy density of 135.8 Wh/kg and a specific power density of 137.0 W/kg at the rate of 1C. At 4C, it maintained a specific energy density of above 100 Wh/kg.

## Sensitivity analysis using design of experiments

**Figure 4:**
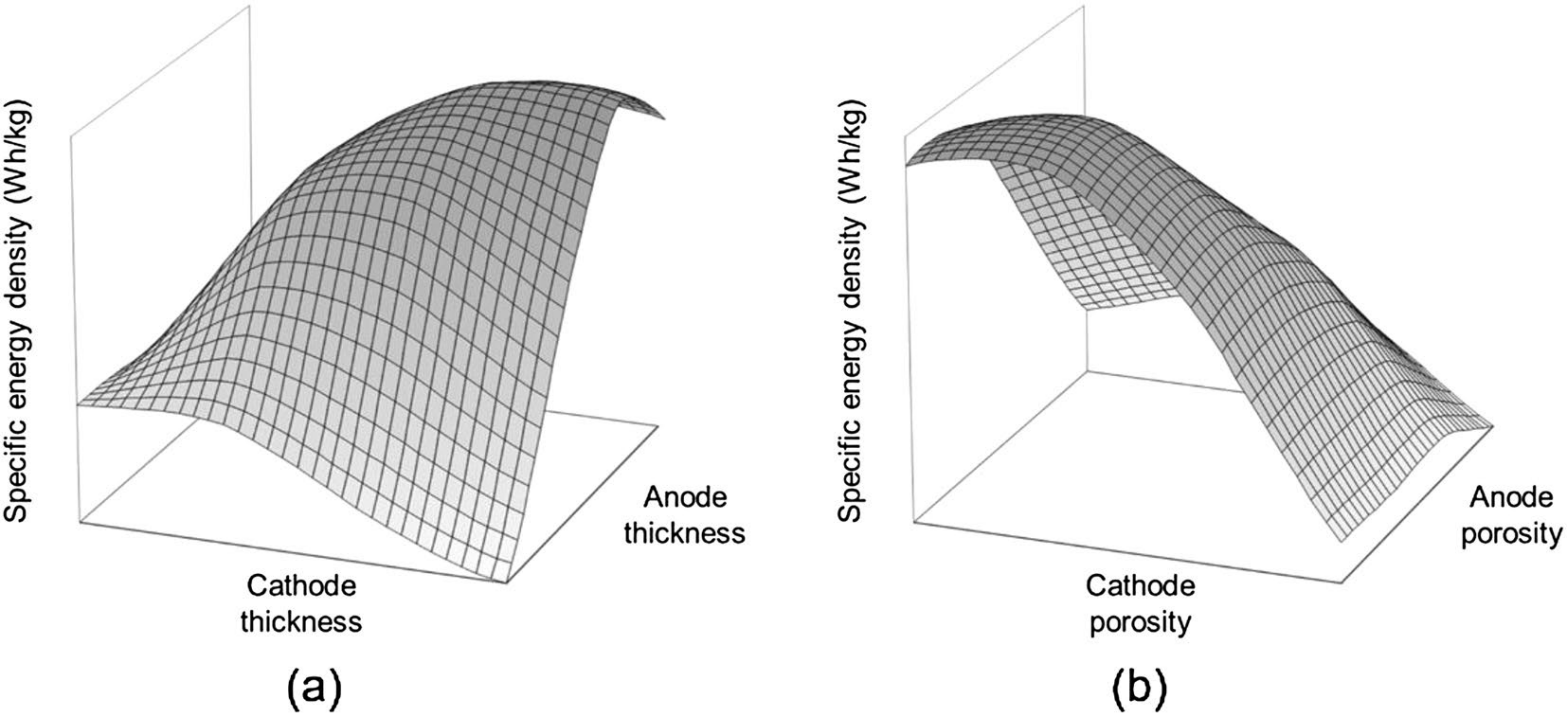
Specific energy density at sampling points of DOE for (**a**) electrode thickness (**b**) electrode porosity.

A DOE was conducted to analyze the sensitivity of the cell design factors that influence the specific power density and specific energy density. The selection of key design variables through sensitivity analysis of DOE is an effective method to reduce unnecessary computational costs and achieve better results in the optimization stage. Eight design factors were selected for the initial cell, including thickness and porosity, and particle size of the anode, cathode, and separator. Diffusivity and conductivity were excluded from the design factors since they are determined during the development of battery materials itself. Table 2 shows the design space of the design factors; they were based on previously reported values^13^. Considering the nonlinearity between the cell’s design factors, specific energy density, and specific power density, sampling points were derived through the 4-level orthogonal array in this design space. Figure 4 shows the relationship of the specific energy density with the thickness and porosity of the anode and cathode for 96 sampling points. This relationship indicates that a high specific energy density can be obtained at a certain thickness ratio of the anode and the cathode. The thickness of the electrode is related to the amount of electrochemically active material inside the cell, indicating that the ratio of the active material of the anode and the cathode must be maintained^13^. As with electrode thickness, porosity is also a factor related to the amount of active material inside the cell. Figure 4b shows that the higher specific energy density is predicted in the electrode with a smaller porosity, and it also implies that the ratio of active material should be considered. This is because, even if the amount of active materials in the positive electrode is large, the entire amount will not react if the quantity of active materials in the negative electrode is small. Both the positive and negative electrodes must have a balanced amount of active material.

**Table 2:** Design factors of DOE and the ranges.

Design factors	Lower bound	Upper bound
Anode thickness (μm)	40	250
Cathode thickness (μm)	40	250
Separator thickness (μm)	10	100
Anode porosity	0.2	0.6
Cathode porosity	0.2	0.6
Separator porosity	0.2	0.6
Anode particle size (μm)	5	20
Cathode particle size (μm)	2	20

A correlation analysis was conducted to analyze the linear relationship between the cell design factors and specific energy density, and cell design factors and specific power density. A correlation analysis is a method of analyzing the linear relationship between factors and responses. The closer the correlation coefficient is to 1, the stronger the positive linear relationship, and the closer to − 1, the stronger the negative linear relationship. Factors with an apparent linear relationship can be excluded from the design variables because the optimal value is at the boundary of the design space. Table 3 shows the results of the correlation analysis. There were no factors with an apparent linear relationship between the cell’s design factors, specific energy density, and specific power density. However, the thicknesses of the anode and cathode displayed a moderate negative linear relationship with the specific power density, suggesting that the thinner the electrode, the higher the specific power density^27^. However, electrode thicknesses cannot be excluded from the design variables because they have a nonlinear relationship with the specific energy density.

**Table 3:** Correlation coefficients of specific energy and specific power for design factors.

	Specific energy density	Specific power density
Anode thickness	0.330	− 0.481
Cathode thickness	0.320	− 0.697
Separator thickness	− 0.108	− 0.162
Anode porosity	− 0.300	0.080
Cathode porosity	− 0.297	0.237
Separator porosity	0.023	− 0.013
Anode particle size	− 0.069	− 0.040
Cathode particle size	− 0.104	− 0.003

Analysis of variance (ANOVA) was conducted to determine the sensitivity of the design factors and accordingly select the design variables for optimization. ANOVA is a method of estimating the impact of the factors on the responses by expressing the distribution of those responses as a sum of squares and separating it by factor^28^. A significance level(α) of 0.05 was used. If P-value was less than 0.05, then it was judged as a factor with statistically significant interaction with the response. Tables 4 and 5 show the ANOVA results. For specific energy density, thicknesses and porosities of the anode and the cathode were determined to be significant factors. For specific power density, thicknesses of the electrodes and the separator and porosity of the cathode were determined to be the significant factors. Through ANOVA, thicknesses of the anode, the cathode, and the separator, and porosities of the anode and the cathode were selected as the design variables.

**Table 4:** ANOVA results of specific energy density.

Factor	DOF	Sum of squares	Mean square	F-value	*P* value
Anode particle size	3	1,529	510	0.419	0.740
Cathode particle size	3	2,308	769	0.633	0.596
Anode porosity	3	19,048	6,349	5.221	0.003
Cathode porosity	3	22,577	7,526	6.189	0.001
Separator porosity	3	963	321	0.264	0.851
Anode thickness	3	37,490	12,497	10.28	< 0.0001
Cathode thickness	3	29,218	9,739	8.009	< 0.0001
Separator thickness	3	3,594	1,198	0.985	0.405
Error	71	86,342	1,216		
Total	95	203,069

**Table 5:** ANOVA results of specific power density.

Factor	DOF	Sum of squares	Mean square	F-value	*P* value
Anode particle size	3	426	142	0.355	0.786
Cathode particle size	3	314	105	0.261	0.853
Anode porosity	3	1,737	579	1.457	0.236
Cathode porosity	3	11,374	3,791	9.473	< 0.0001
Separator porosity	3	523	174	0.436	0.728
Anode thickness	3	48,482	16,161	40.38	< 0.0001
Cathode thickness	3	100,905	33,635	84.04	< 0.0001
Separator thickness	3	7,766	2,589	6.468	0.001
Error	71	28,416	400		
Total	95	199,943			

## Specific energy density optimization of lithium-ion battery cell

### Formulation of optimization problem

To improve the specific energy density while maintaining the initial power performance, ± 1% of the initial specific power density was used as a constraint. The design variables included anode thickness (x_1_), cathode thickness (x_2_), separator thickness (x_3_), anode porosity (x_4_), and cathode porosity (x_5_) that were selected through the sensitivity analysis of DOE. The upper and lower ranges of the design variables are shown in Table 2. At a rate of 1C, the battery cell was subjected to a constant current discharge and a cutoff voltage of 3.0 V.

9 $$\begin{aligned} & Find\quad \quad {:}\quad x_{k} , \ldots \ldots ,k = 1, 2, 3, 4, 5 \\ & maximize{:} \quad E = \frac{1}{{M_{cell} }}\mathop \smallint \limits_{0}^{{t_{end} }} V_{cell} \cdot i_{app} dt \\ & subject\,to\quad {:}\quad 135.6\,{\text{W/kg}} < P < 138.4\,{\text{W/kg}} \\ & \quad \quad \quad \quad \quad x_{lower,k} \le x_{k} \le x_{upper,k} \\ \end{aligned}$$

### PQRSM algorithm

**Figure 5:**
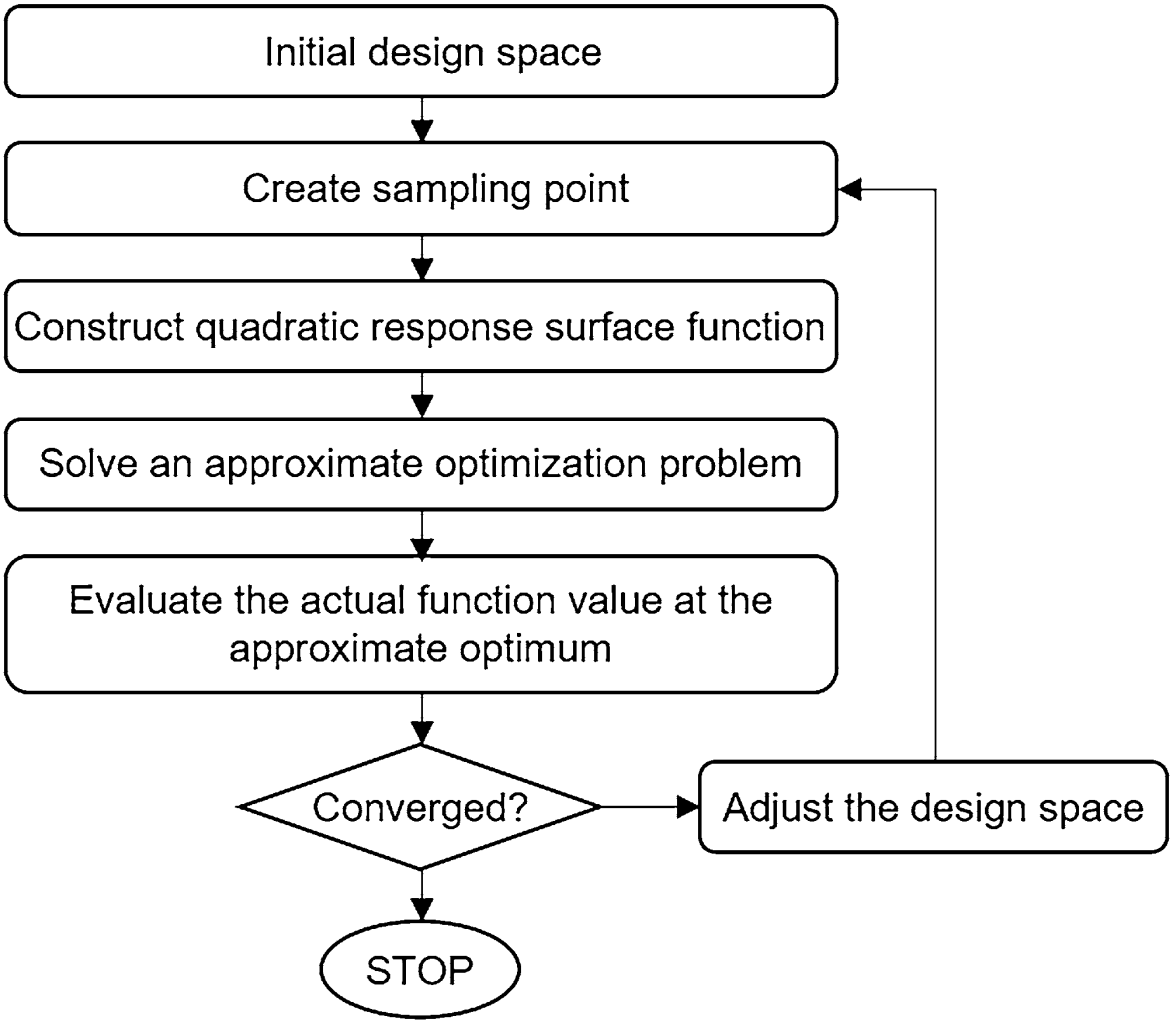
Flowchart of the PQRSM optimization process.

The PQRSM algorithm is one of the SAO techniques that uses a quadratic response surface model. Unlike gradient-based algorithms, the gradient is not needed, thus no complex computations are required. This can also be applied to problems with numerical noise and is effective for nonlinear problems^20,21^. Moreover, the trust region algorithm, which guarantees weak global convergence, is applied. Consequently, the probability of convergence on the local optimum is low^22,23^. The PQRSM algorithm generates a full quadratic response surface model which satisfies rotatability with each iteration with 2n + 1 sampling points and conducts an approximate optimization using this response surface model. Figure 5 shows the PQRSM optimization procedure. First, one center point and 2n axial points in the design space are selected as the sampling points. These points are then used to generate the quadratic response surface model and the approximate optimization is conducted in the initial trust region. The actual function value is then calculated from this approximate optimum and the convergence criteria are evaluated. If the convergence criteria are not satisfied, this process of optimization is repeated until a new trust region is created and converged.

## Results and discussion

**Figure 6:**
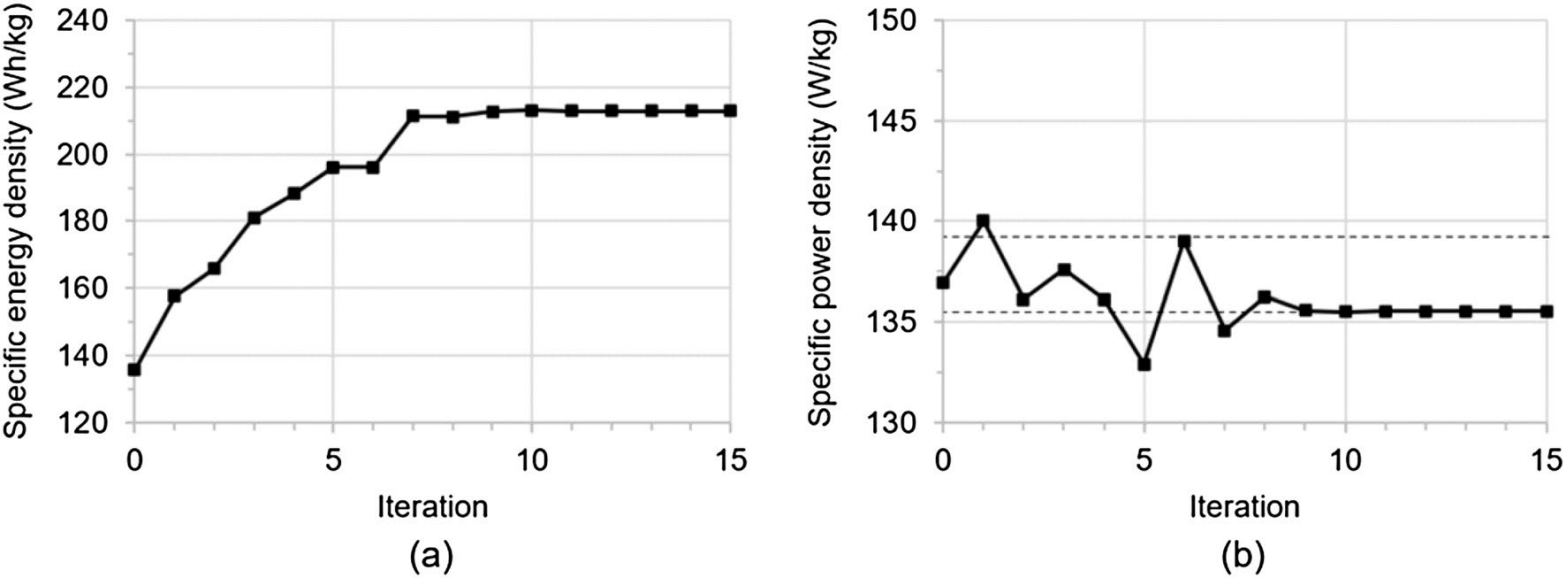
Convergence history of optimization of (**a**) objective function: the specific energy density and (**b**) constraints: the specific power density.

This study shows the potential of PQRSM based optimization to design cells with maximized energy density while maintaining specific power requirements. As a result of optimization, the specific energy density increased by 56.8% and the specific power density decreased by 1.02% while satisfying the constraints. Figure 6 shows the convergence history of optimization. Optimization converged after 15 iterations. Table 6 shows changes in the design variables, the objective function, and the constraints after optimization.

**Table 6:** Comparison of objective function, constraints and design variables between initial cell and optimized cell.

Objective function and constraints	Initial cell	Optimized cell	Rate of change (%)
Specific energy density (Wh/kg)	135.8	212.9	56.8
Specific power density (W/kg)	137.0	135.6	− 1.02
Design variables			
Anode thickness (μm)	100	118.9	18.9
Cathode thickness (μm)	174	130	− 25.3
Separator thickness (μm)	52	10	− 80.8
Anode porosity	0.357	0.2	− 44.0
Cathode porosity	0.444	0.2	− 55.0

**Figure 7:**
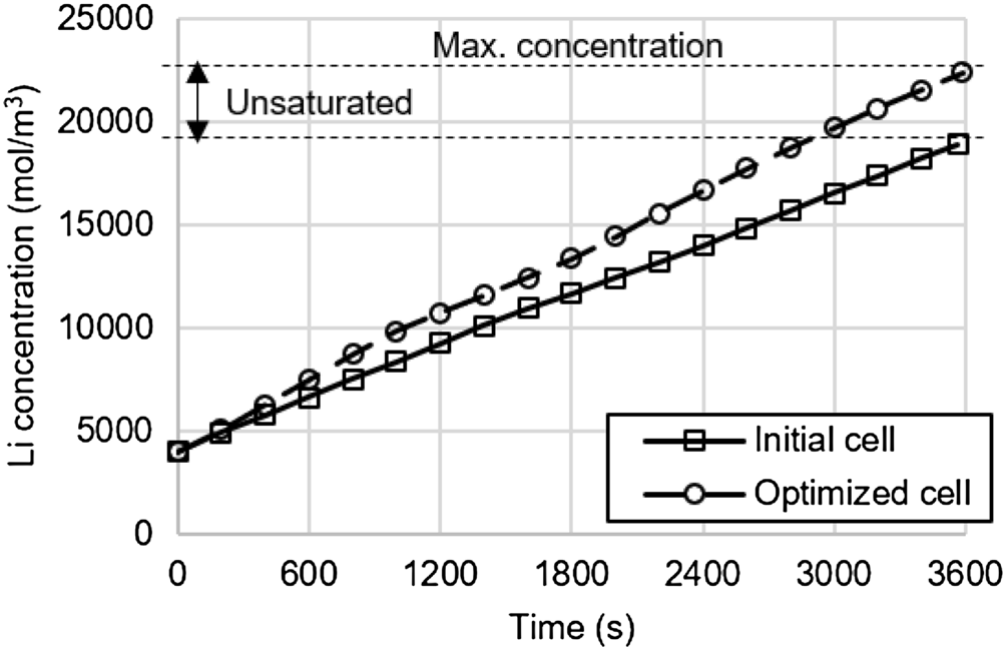
Variation of Li concentration in the solid phase of cathode for initial and optimized cells during 1C discharge.

The thickness ratio of the anode and the cathode changed from the initial value of 1.74 to 1.09, which improved the negative/positive capacity ratio of the cell from the initial value of 0.86 to 1.09. An effective thickness ratio between the anode and cathode is determined by the ratio of active materials of the two electrodes for a balanced electrochemical reaction. Figure 7 illustrates the changes in concentration of the cathode solid phase of the initial cell and the optimized cell during 1C discharge. The cathode of the initial cell was unsaturated at the cut-off voltage due to the lack of active material at the anode. But the optimized cell was improved to the extent that both electrodes have a balanced amount of active material, and the cathode became saturated at the end of the discharge, thus improving the specific energy density of the cell. In addition, reducing the cathode thickness also reduced the electrode resistance, thereby enhancing the specific energy density at higher power^27^. The separator thicknesses converged on the lower boundary. The separator is not involved in the electrochemical reaction; thick separators increase the distance of the ions and increase the mass, thus decreasing the specific energy density. Porosity is a design variable that changes the amount of active material and electrolytes inside the electrode and influences the mass transfer capability of the ions. The porosity of the two electrodes decreased to the lower boundary. Reducing the porosity increased the amount of active material inside the electrode, thus increasing the theoretical energy. However, this reduces the amount of electrolyte, thereby decreasing the mass transfer capability of the ions at high powers^29,30^. The specific energy density of the cell was enhanced by effectively increasing the amount of active material while maintaining the mass transfer capability of the ions at the target power.

**Figure 8:**
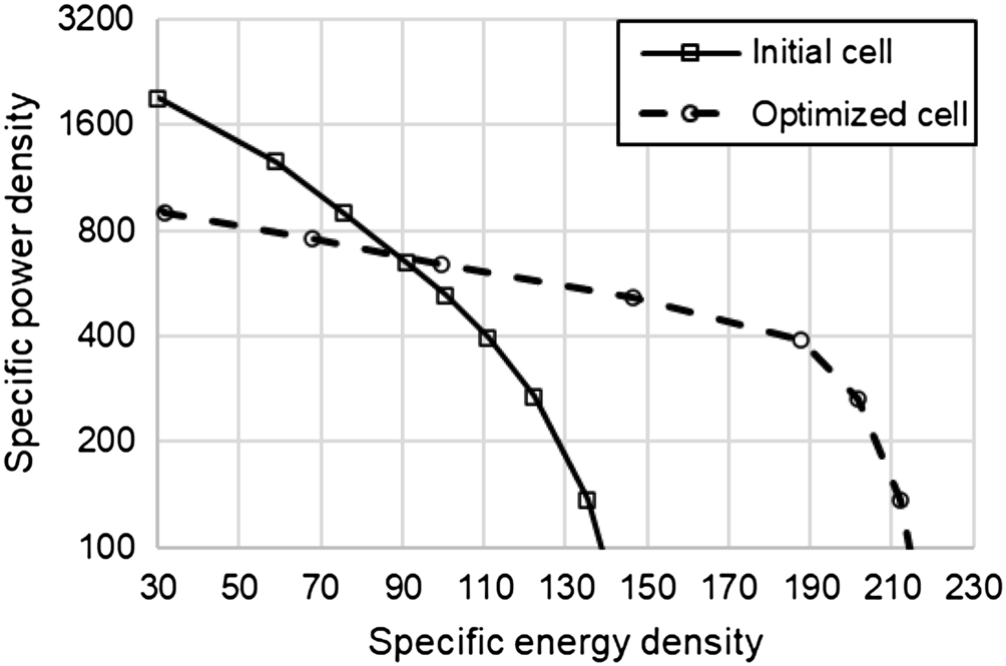
Specific energy density versus specific power density of initial cell and optimized cell.

**Figure 9:**
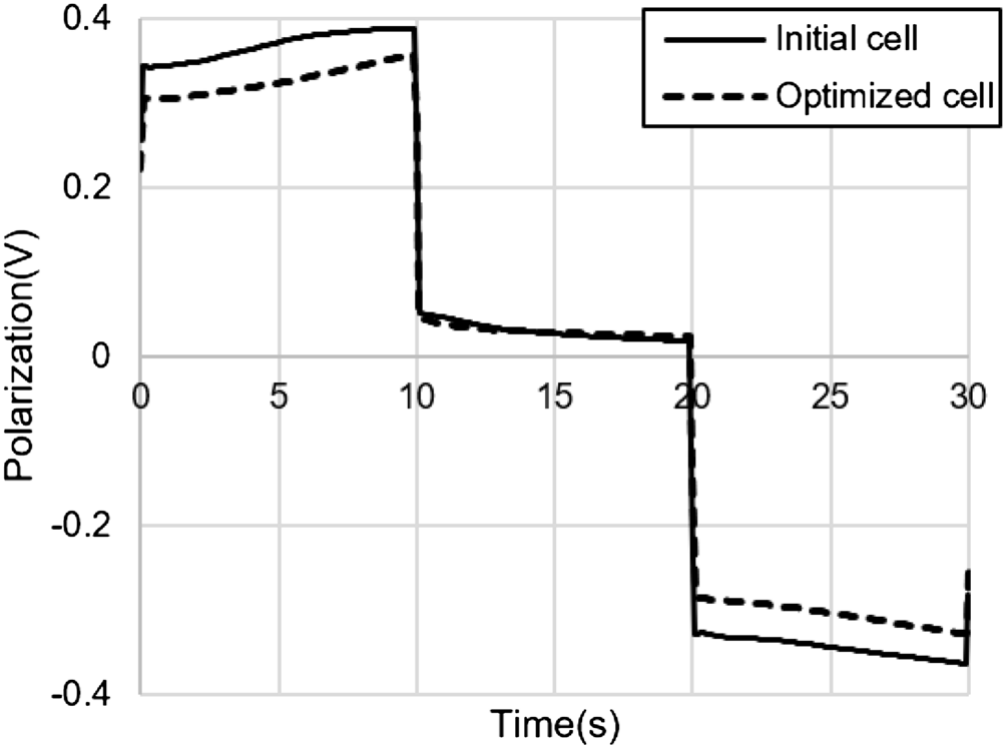
Polarization of initial cell and optimized cell in HPPC test.

Figure 8 is a graph of specific energy density vs. specific power density of the initial and optimized cells. At a specific power density of 600 W/kg and below, the optimized cell showed a higher specific energy density performance than the initial cell. An HPPC analysis was conducted to confirm the polarization phenomenon of the cell. The battery was discharged at 10C for 10 s and recharged at 10C after 10 s of relaxation. Figure 9 illustrates the results. As a result of optimization, the polarization phenomenon was reduced by 11.5% compared to the initial model. This result demonstrates that the theoretical energy effectively increased without increasing the polarization phenomenon despite a reduction in porosity. On the other hand, changes in LIB cell design variables affect cycling stability of electrodes and thus affect the life cycle of LIB. For more practical LIB optimization in the future, we should include degradation models that consider capacity fade mechanisms.

## Conclusions

In this study, optimization was conducted to maximize the specific energy density of the LIB using DOE and PQRSM, which guarantees weak global convergence and does not need to calculate gradients. First, DOE was performed to select the design variables, the sampling points were derived through the 4-level orthogonal array, and the sensitivity was analyzed through correlation analysis and ANOVA. The correlation analysis confirmed that no factors had any apparent linear relationship and that the thickness of the electrode had a moderate negative linear relationship with specific power density. Through ANOVA, cathode thickness, anode thickness, separator thickness, cathode porosity, and anode porosity were selected as the design variables, which are factors influencing the specific energy density and specific power density.

Then, The LIB was optimized to maximize the specific energy density while maintaining the specific power density using PQRSM. The result of the optimization revealed that the specific energy density was improved by 56.8% while satisfying the constraints. The improvement of the thickness ratio of the electrode improved the ratio of active material and the reduction of porosity increased the amount of active material without disturbing the mass transfer capability at the target power. An HPPC analysis was conducted on the initial and optimized cells, which demonstrated an 11.5% reduction in energy loss due to the polarization phenomenon. This study verified that PQRSM-based optimization is effective for designing high-capacity batteries.

## List of symbols

*a*: Ion number
*ϕ*: Electrical potential (V)
*η*: Local surface overpotential (V)
*ρ*: Density (kg/m^3^)
*ε*: Porosity
*α*: Significance level
*A*_*cell*_: Cross-sectional area of cell
*c*: Li concentration (mol/m^3^)
*D*: Diffusivity (m^2^/s)
*E*_*cell*_: Specific energy density (Wh/kg)
*P*_*cell*_: Specific power density (W/kg)
*f*_±_: Average molar activity coefficient
*F*: Faraday’s constant, 96,487 (C/mol)
*i*: Current density (A/m^2^)
*j*_*n*_: Local current density (A/m^2^)
*k*: Electronic conductivity (S/m)
*M*_*cell*_: Mass of cell (kg)
*N*_0_: Li^+^ flux (mol/m^2^s)
*r*: Radial distance from the center of electrode’s active particle (μm)
*R*_*c*_: Gas constant, 8.314 (J/(mol K))
*t*: Thickness (m)
*t*_+_: Transport number of Li^+^
*T*: Absolute temperature (K)
*V*_*cell*_: Electric potential of cell

## Subscripts and superscripts

app: Applied
*eff*: Effective value
*el*: Electrolyte
*neg*: Negative electrode
*pos*: Positive electrode
*sep*: Separator
1: Solid phase
2: Liquid phase
